# Psychotropic drugs and driving: prevalence and types

**DOI:** 10.1186/1744-859X-13-14

**Published:** 2014-05-08

**Authors:** Francisco Alonso, Cristina Esteban, Luis Montoro, Francisco Tortosa

**Affiliations:** 1 DATS (Development and Advising in Traffic Safety) Research Group, INTRAS (Research Institute on Traffic and Road Safety), University of Valencia, Serpis 29, Valencia 46022, Spain; 2 FACTHUM.lab (Human Factor and Road Safety), INTRAS (University Research Institute on Traffic and Road Safety), University of Valencia, Serpis 29, Valencia 46022, Spain; 3 PRECOVIR (Prevention of Risk Behavior on the Road), INTRAS (Research Institute on Traffic and Road Safety), University of Valencia, Serpis 29, Valencia 46022, Spain

**Keywords:** Drivers, Road safety, Medicines, Psychotropic drugs, Epidemiology, Public health

## Abstract

**Background:**

Some psychotropic medications (e.g., benzodiazepines, sedative antidepressants, etc.) may impair cognitive and psychomotor functions and, therefore, endanger traffic safety (Ravera, Br J Clin Pharmacol, 72(3):505–513, 2011). They affect detection, registration, and information processing, problem solving, and decision-making processes, and they also affect emotional and social aspects. The objective of this research was to clarify three closely related issues that are significant for traffic safety: the prevalence of psychotropic drugs on driving, the most frequently used psychotropic drugs to treat depression, anxiety, insomnia, or any tranquilizers (whether it is a medical prescription or self-medication), and finally, provide a further understanding of the socio-demographic and psycho-social characteristics of drivers related to the psychotropic drugs consumption in Spain.

**Methods:**

A sample of 1,200 Spanish drivers ranging from 18 to 64 years was used, 666 men and 534 women were asked to answer a questionnaire composed by a set of questions structured in different sections. The only selection criteria were to be in possession of any type of driving license for vehicles other than motorcycles and drive frequently.

**Results:**

The results showed that 15% of the participants were consuming psychotropic drugs to treat depressive disorders, anxiety disorders, insomnia, or tranquilizers; 13.5% were using drugs to treat one of these disorders; while 1.5% used them for several of these disorders. A 2.5% of drivers were using medicines to treat depression, 2.6% to treat anxiety, and 3.7% to treat insomnia. The 8.3% of those drivers who were not using any drugs to treat these three disorders were occasionally using some type of tranquilizers. Benzodiazepines and selective serotonin reuptake inhibitors (SSRIs) were the most used type of medicines among drivers. Benzodiazepines were the most used medicines to treat anxiety, while SSRIs were the most used to treat depression, 56.5% and 43.5%, respectively.

**Conclusions:**

Measures can be developed to reduce traffic accidents caused by the effects of these drugs; however, this will only be possible once the drivers and the use of these drugs are understood. Health care professionals and patients should be properly informed about the potential effects of some psychotropic medications on driving abilities considering individual and group differences.

## Introduction

Many psychiatric disorders may present problems with driving [1,2]. However, decisions regarding fitness to drive on psychiatric grounds may be difficult because of the subjective nature of the symptoms and difficulty in prediction of disturbed behavior.

Most mental illnesses tend to reduce activity and interest and therefore possibly the use of a car [3]. However, in a study [4], the majority of the psychiatric patients studied had a driving license and were driving on a daily basis, and 79.5% of them failed to pass the required tests (general driving license tests obligatory to obtain the license according to the Spanish Medical and Psychotechnical Exam Model). The most worrying finding was that ten participants out of those who were driving were professional drivers (and only two of them passed the tests). Moreover, psychiatric drug treatments may cause changes in perception, information processing and integration, and psychomotor activity that may disturb and/or interfere with the ability to drive safely [5,6].

When it comes to traffic, drugs are one of the many factors that may affect the ability to drive safely, even though they are not the main cause of traffic accidents [7]. Because of that, the high number of drivers who are under treatment is an aspect of great relevance that affects road safety. In this sense, research has shown that taking psychotropic drugs can cause a higher risk of getting involved in a traffic accident [8].

The responsibility of psychotropic drugs as a cause of road traffic accidents remains difficult to evaluate with precision. Different studies performed in many countries have provided a certain precision in relation to the percentage of injured drivers whose blood contained psychotropic substances (8% to 10% according to studies). On the other hand, it is practically impossible to really know either these substances were or were not the cause of the accidents because underlying or associated pathologies may equally create problems such as lack of attention and other vigilance deficits. There is also a possibility of suicidal or aggressive tendencies [9].

A certain number of circadian and other chronobiological parameters also complicate the problem since the schedule (hour) as well as the day of the week or even the season can considerably modify vigilance and reaction time. Available medications able to create such problems are numerous, and their mechanisms of action are varied. They may affect vision, impulsiveness, and vigilance. They can act either by direct mechanisms of sedation or, on the contrary, by raising inhibition through secondary mechanisms: delay in drug elimination or causing insomnia. For the most part, incriminated medications belong to the different classes of sedative medicines: benzodiazepines, antiepileptics, some antihistaminic agents, some antidepressants, some thymoregulators and some antihypertensives. If it appears methodologically impossible that research could ever precisely quantify the share of responsibility of psychotropic drugs in causing road traffic accidents, this relation remains highly probable [9].

It has been known for many years that the use of psychotropic substances, such as alcohol, sedatives, anxiolytics, antidepressants, or illicit drugs, has a negative effect on the ability to drive [10].

Several classes of drugs, including amphetamines, antihistamines, cannabis, hypnotics, tranquilizers, and tricyclic antidepressants, have been shown to impair driving skills in laboratory tests and driver-simulation studies [11-13].

It is also apparent that drugs in combination with alcohol, and multiple drugs, present an even greater risk. Drug driving is a significant problem, both in terms of a general public health issue and as a specific concern for drug users [14]. In fact, either alone or in combination, alcohol and psychoactive substances increase the risk of having a traffic accident [10,15-17].

Specifically, using antidepressants, benzodiazepines, and sleeping pills known as Z-drugs are all associated with a significantly increased risk of car accidents, according to a study about psychotropic drugs linked to increased car accidents. It was concluded that participants should be properly informed of the potential risks associated with the use of these medicines [1,18].

The Driving under the Influence of Drugs, Alcohol and Medicines (DRUID) project was an integrative effort to reduce the danger of alcohol, illicit drugs, and medicines in traffic. Regarding prevalence of medicinal drugs in European countries, the study conclusions were as follows:

• Illicit drugs are most prevalent among the population in the Southern European countries whereas medicines are most prevalent in the Nordic countries (Denmark, Norway, Sweden, Finland).

• EU mean, all psychoactive medicines: 1.4%; range across countries: 0.17%–2.99%).

• The prevalence rate for medicines in Spain was 1.6%.

• Benzodiazepines were the most prevalent medicinal drug in traffic. Z-drugs were less prevalent. However, considerable differences between countries were present.

• The medicinal drugs in general mainly detected among older female drivers during daytime hours.

• More prevalence in men, in 35–49 years old, roads (not urban), weekend and holiday in daytime (working day in dark).

The epidemiology reveals a low risk for injury (1.5–3) and a higher fatality risk (5–7) for the group of ‘benzodiazepines and Z-drugs’. The risk of medicinal opioids is high for injury (5–8) but lower for a fatality (5) [19,20].

Usually, multiple administration of a psychoactive substance to naïve subjects leads to adaptation after some time of use. This means that after some days of use of a psychoactive substance, the degree of performance impairment decreases. The degree of adaption depends on many factors, especially the dose and the frequency of use.

The condition in patients is by far even more complex than the situation during adaption of healthy subjects because the disease itself might have impairing effects on performance that might be decreased by the medicament itself. Thus, the impairing effects are determined by an interaction of these factors. For further discussion of this problem, see [21].

### Study framework

Connections between traffic and illnesses are strong and complex, and they are beyond the existing relation of the ability to drive and the probability of being involved in a traffic crash. Health, beyond the absence of any illness, entails the full self-perceived biopsychosocial state of well-being [22]. From this approach, road health has to be treated from a comprehensive perspective, i.e., taking into account the biological, psychological, and social aspects [23,24]. Moreover, it is important to understand the health-related causes of drivers that may impair driving in order to prevent motor vehicle collisions and, also important, for drivers to be aware of this risk. So, this is why the framework of this article was a large-scale project on ‘road safety and health’ to raise people's awareness regarding this matter [23-25].

This global research on health and driving used a questionnaire made up of a set of items in different sections. First of all, the questionnaire was used to collect socio-demographic and psychosocial data of drivers.

There were also subsections to collect information related to four areas: ‘subjective incidence of health in driving’ , ‘drivers' psychological state (condition)’ (including symptom scales for depression, fatigue, anxiety, and daily and work stress), ‘medication and driving’ , and ‘the system of selection of drivers’ (view and proposal).

The study described in this article is based on the data found in the section ‘medication and driving’. In the section of the questionnaire, participants were asked whether they were under pharmacological treatment for anxiety, depression, or insomnia. If not, they were asked whether they were using tranquilizers occasionally. If so, they were asked to state the type of medicine used and whether it had been prescribed by a doctor. In addition, in order to understand the perception of drivers about how medicines treat depression, anxiety, insomnia, or tranquilizers affect driving, they were asked whether they thought that these medicines could affect their driving. It was also interesting to learn about their perception regarding the amount of information they had about medicine use and how did they learn about the influence of medicines on driving. The relationship between different treatments, type of medicine, and medical prescription was also analyzed in the global research [25].

### Objective

The specific objectives of this survey were as follows:

1. To know the prevalence of psychotropic drugs (drugs to treat depression, anxiety, insomnia, or other tranquilizers) in drivers.

2. To identify the most used type of drug

3. Provide a further understanding of the socio-demographic and psychosocial characteristics of drivers related to the psychotropic drugs consumption.

In general terms, these aspects will be used to design interventions and to increase road safety.

## Method

### Participants

Participants were part of a wide-ranging research on different aspects of health that affect driving. The sample used was composed of 1,200 Spanish drivers ranging from 18 to 64 years, 666 men (56%) and 534 women (44%). The starting sample size was proportional by quota to the Spanish population segments of age and gender. The number of participants represents an error margin for the general data of ±2.9 with a 95% confidence interval in the most unfavorable case of *p* = *q* = 50%.

Drivers completed a telephone-based survey. Interviews were completed for 1,200 drivers, and the response rate was 92.8%; as it was a survey dealing with social matters, the vast majority of people wanted to collaborate. There were 93 (7.2%) people who did not want to participate in the interview.

### Procedure and design

The survey was conducted by telephone. A national telephone household sample was constructed using random digit dialing. Each household was screened to determine the number of adult (age 18 or older) drivers in the household. The only selection criteria were to be in possession of any type of driving license for vehicles other than motorcycles and drive frequently. One eligible driver was systematically selected in each eligible household by the interviewers. The survey was conducted using the computer-assisted telephone interviewing (CATI) system to reduce interview length and minimize recording errors, guaranteeing at all times the anonymity of the participants, and stressing on the fact that the data would only be used for statistical and research purposes. The importance of answering honestly to all the arisen questions was emphasized, as well as the non-existence of wrong or right answers.

In this article, the data obtained was analyzed in the questions as follows: ‘Are you currently under pharmacological treatment for any of these ailments? Anxiety, depression or insomnia’. The participants answered ‘yes’ or ‘no’ for each disorder. If their answer was ‘no’ for all of them, they were asked: ‘Do you occasionally use any drug or pill to relax?’. If their answer was ‘yes’ , in anyone, they were asked to say the type of medicine they used.

First of all, the questionnaire was used to collect data to establish a profile of the interviewed as a driver, with the aim of detecting the distinguishing characteristics that define their inclusion into a certain group(s). These variables mainly focused on socio-demographic and psychosocial characteristics grouped in the following subsections.

#### Demographic variables

The following are the demographic variables:

• Sex (man or woman)

• Age (grouped in 18–25, 26–35, 36–45, 46–55, 56–65, over 65)

• Population size where they live (strata considered are as follows: in less than 10,000; from 10,001 to 20,000; 20,001 to 100,000; 100,001 to 500,000; and more than 500,000)

• Work activity (grouped in active, not active, housework)

• Profession (grouped in self-employed, management, other employees employed)

• Working time (day, night, and shifts)

#### Driving habits

The following are the driving habits:

• Day/night driving (by day, by night, either).

• Continuous driving by journey (grouped in less than 1 h; for 1 to 2 h; 2 or more hours).

• Type of road more frequently used for driving (grouped in urban zones; conventional roads; highway).

• Type of vehicle used (grouped in utilitarian vehicles—conventional cars, sports cars, and family—and commercial or transportation vehicles such as vans, trucks, buses, etc.

• Risk exposure. To determine the level of risk exposure of the driver interviewed, both the average miles driven per year as well as the frequency driven were taken into account. The combination of both variables have led to a classification of drivers in five groups:

Exposure to very low risk: includes mainly sporadic drivers (low frequency and/or few km/year).

Exposure to low risk: includes drivers who made sporadic but long trips (e.g., vacation) or even those who drive frequently but made very few kilometers per year.

Average risk exposure: includes regular drivers who do not average many kilometers per year as their movements are not excessively long (e.g., urban trips or weekend outings).

Exposure to high risk: includes the usual drivers averaging significant kilometers per year because their movements are relatively long (i.e., their commute to and from work).

Exposure to very high risk, including those who drive frequently and that in turn make many kilometers per year (e.g., professional drivers, commercial, delivery, etc.).

• Reason for driving. Grouped in *itinere* (on the way to or from work), during work, leisure, and/or personal, regardless labor or leisure).

#### Experience/risk

The following are the experiences/risks:

• Years of driving experience. Experience has been defined as the time that the respondent has been driving on a regular basis. This variable is complementary to the risk exposure, since both variables are an indicator of learning situations (both positive and negative) that the respondent has been able to experience in their driving history. (Grouped in less than 1 year, 1–2 years, 3–10 years, 11–20, 21–30, over 30 years).

• Risky behavior. The risky taking is calculated by five items. The objective of this set of items is to rate drivers for certain risk behaviors (exceeding speed limits and not keep a safe distance, making a rushed or improper pass, driving after drinking alcohol, using a mobile while driving without using a hands-free device). For each behavior considered have applied the classification criteria of risk-no risk used in the study SARTRE 3 [26], depending on how often they engaged in these behaviors. Taking these criteria into account, drivers have been classified into three groups:

‘No Risk’ group: drivers that have not been classified in any of the risk behaviors considered.

‘Medium risk’ group: drivers who have been classified in one or two risk behaviors considered.

‘High risk’ group: drivers of risk are classified in more than one of the considered behaviors.

• Traffic violations. Number of penalties received in the last 3 years, excluding parking offenses (none, one, more than one penalty).

• Crash history. Number of accidents occurring throughout a driver's life, focusing primarily on accidents suffered as a conductor (none, one, more than one accident).

Once the data was obtained, the relevant statistical analyses were carried out with the Statistical Package for the Social Sciences (SPSS).

## Results

The results showed that 15% of the participants were consuming psychotropic drugs, to treat depressive disorders, anxiety disorders, insomnia disorders, or tranquilizers; 13.5% were using drugs to treat one of these disorders; while 1.5% used them for several of these disorders.

**Figure 1:**
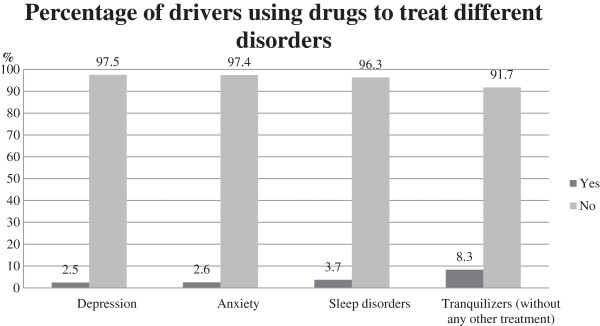
Percentage distribution of drivers using drugs to treat depression, anxiety, stress, or tranquilizers.

A 2.5% of drivers were using medicines to treat depression, 2.6% to treat anxiety, and 3.7% to treat insomnia. The 8.3% of those drivers who were not using any drugs to treat these three disorders were occasionally using some type of tranquilizers (7.7% of the total drivers interviewed) (Figure 1).

There were statistically significant differences for gender (*x*^2^ = 8.101, *p* ≤ 0.005), and age (*x*^2^ = 13.666, *p* ≤ 0.05) among the 2.5% of drivers that were being treated for depression; women (70% of the interviewed drivers under treatment for depression) and adults (46–55) used drugs more often than any other age group. Table 1 shows the frequency and percentage of people *with* or *without depression medication* classified according to their gender or age.

**Table 1:** Frequency and percentage of people with or without depression medication classified according to their gender or age

		**With depression medication**		**Without depression medication**	
		**Frequency (*n*** **= 30)**	**Percentage**	**Frequency (*n*** **= 1170)**	**Percentage**
Gender	Women	21	3.9	513	96.1
	Men	9	1.4	657	98.6
Age	18–25	0	0	125	100
	26–35	4	1.5	261	98.5
	36–45	11	3.2	335	96.8
	46–55	12	5.2	220	94.8
	56–65	3	1.7	169	98.3
	> 65	0	0	60	100

Regarding anxiety, adults ranging from 36 to 45 years (*x*^2^ = 13.306, *p* ≤ 0.05) were the group that most used anxiety medication, even though the percentage of drivers that were being treated for this disorder was small (2.6%). However, it was not possible to establish a significant relationship between gender and drug use to treat this disorder. Table 2 shows the frequency and percentage of people *with* or *without anxiety medication* classified according to their gender or age.

**Table 2:** Frequency and percentage of people with or without anxiety medication classified according to their gender or age

		**With anxiety medication**		**Without anxiety medication**	
		**Frequency (*n*** **= 31)**	**Percentage**	**Frequency (*n*** **= 1169)**	**Percentage**
Gender	Women	19	3.6	515	96.4
	Men	12	1.8	654	98.2
Age	18–25	2	1.6	123	98.4
	26–35	3	1.1	262	98.9
	36–45	17	4.9	329	95.1
	46–55	7	3.0	225	97.0
	56–65	2	1.2	170	98.8
	> 65	0	0	60	100

By contrast, adults ranging from 56–65 years took more drugs to treat insomnia, even though the differences in these groups did not reach the level of importance required (*x*^2^ = 10.229, *p* ≤ 0.07). In this case, it was not possible to establish a significant relationship between gender and drug use to treat this disorder. Table 3 shows the frequency and percentage of each gender and each age group *with* or *without insomnia medication*.

**Table 3:** **Frequency and percentage of each gender and each age group *with *** **or *without insomnia medication***

		**With insomnia medication**		**Without insomnia medication**	
		**Frequency (*n*** **= 44)**	**Percentage**	**Frequency (*n*** **= 1156)**	**Percentage**
Gender	Women	22	4.1	512	95.9
	Men	22	3.3	644	96.7
Age	18–25	2	1.6	123	98.4
	26–35	5	1.9	260	98.1
	36–45	11	3.2	335	96.8
	46–55	13	5.6	219	94.4
	56–65	11	6.4	161	93.6
	> 65	2	3,3	58	96.7

Likewise, tranquilizers are used by 8.3% of the drivers who were not using drugs to treat depression, anxiety, or insomnia; adults ranging from 56–65 used these drugs more often. In this case, it was not possible to establish a significant relationship between gender and drug use. Therefore, information in Table 4 shows the frequency and percentage of different gender and age groups *with* or *without tranquilizers*.

**Table 4:** **Frequency and percentage of different gender and age groups *with *** **or *without tranquilizers***

		**With tranquilizers**		**Without tranquilizers**	
		**Frequency (*n*** **= 92)**	**Percentage**	**Frequency (*n*** **= 1021)**	**Percentage**
Gender	Women	41	8.5	444	91.5
	Men	51	8.1	577	91.9
Age	18–25	6	5.0	115	95.0
	26–35	21	8.3	233	91.7
	36–45	23	7.3	294	92.7
	46–55	17	8.3	189	91.7
	56–65	20	12.7	137	87.3
	> 65	5	8.6	53	91.4

Regarding the significative relationship between variables related to driving, experienced drivers, 11–20 years of experience (4%, *n* = 12) and 21–30 years of experience (4.2%, *n* = 12), and drivers who drive ‘regardless labor or leisure’ (4.3%, *n* = 16) have a higher percentage of drivers under treatment for depression. On the other hand, men (98.6%, *n* = 657), young people, 18–25 years old (100%, *n* = 125), drivers with 3–10 years of experience (99.3%, *n* = 275), or with more than 30 years (99.5%, *n* = 214), those who use their vehicle for ‘personal and/or leisure’ (98.6%, *n* = 419) and during work (98.7%, *n* = 220), and those who drive in daylight (98%, *n* = 887) are the groups in which there are high percentages of drivers who are not under treatment for depression. It was not possible to establish a significant relationship between size of town, risk exposure, type of vehicle, hours of non-stop driving in usual commutes, most frequently used type of road, crashes, sanctions over the last 3 years (except parking tickets), risk assumed, working status, profession, or work schedule.

Regarding anxiety, drivers who live in towns with more than 500,000 people (6.1%, *n* = 12), and adults ranging from 36–45 years (4.9%, *n* = 17) are the groups with a bigger number of people under treatment for this disorder, even though the total percentage of drivers for it is small (2.5%). In contrast, active workers (98.1%, *n* = 819), and people living in towns with no more than 10,000 people stated (99.3%, *n* = 217) they were using medicines for this disorder less frequently.

It was not possible to establish a significant relationship between age, type of vehicle, risk exposure, driving experience, reasons for the journey, daylight/night driving, hours of non-stop driving in daily commutes, most frequently type of road, crashes, sanctions over the last 3 years (except parking tickets), risk assumed, and profession.

**Figure 2:**
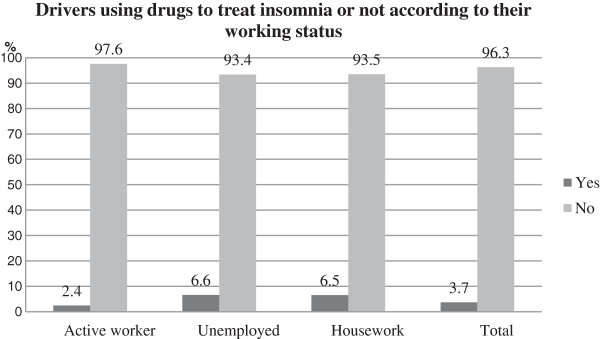
Drivers with insomnia according to their working status, and comparison with the general distribution.

On the other hand, the percentage of people using medicines to treat insomnia is slightly higher (3.7%). The number of people ranging from 36–45 years (3.2%, *n* = 11), 46–55 years (5.6%, *n* = 13), 56–65 years (6.4%, *n* = 11) are the groups with a bigger number of people under treatment for this disorder. The percentage of active workers using medicines to treat insomnia is 2.4%, *n* = 20, while this percentage increases until 6.6%, *n* = 18, for the group of unemployed people (Figure 2).

It was not possible to establish a significant relationship between size of town, gender, risk exposure, type of vehicle, driving experience, reasons for the journey, daylight/night driving, hours of non-stop driving in daily commutes, most frequent type of road, crashes, sanctions over the last 3 years (except parking tickets), risk assumed, profession, and work schedule.

Finally, it is important to remember that 8.3% of drivers (only taking into account those who do not use medicines to treat depression, anxiety or insomnia) stated they were sometimes using tranquilizers. The use of these medicines is more frequent in drivers living in towns of 10,000–20,000 people (12.8%, *n* = 16) and in drivers ranging from 56–65 years (12.7%, *n* = 20).

**Figure 3:**
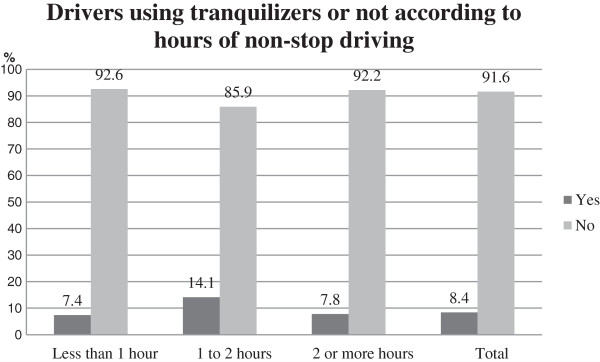
Drivers using tranquilizers or not according ‘hours non-stop driving’ comparing with the general distribution.

Regarding the variables related to driving, drivers with an average risk exposure (12.1%, *n* = 29), those drivers with more than 30 years of driving experience (13.9%, *n* = 28), and those who drive non-stop for 1 or 2 h (14.1%, *n* = 23, of drivers in this last group used tranquilizers) were the groups that used tranquilizers more frequently (Figure 3). In addition, they were drivers who were sanctioned over the last 3 years (except parking tickets) (13.3%, *n* = 21), who were unemployed (13.2%, *n* = 32), or who worked on their own (10.7%, *n* = 17).

However, the probability of using tranquilizers was smaller for those drivers who were active workers (93.1%, *n* = 733), less than 1 h in the continuous driving by journey (92.6%, *n* = 793).

It was not possible to establish a relationship between the use of tranquilizers and gender, type of vehicle, reasons for the journey, type of road, daylight/night driving, crashes, risk assumed, or work schedule.

**Figure 4:**
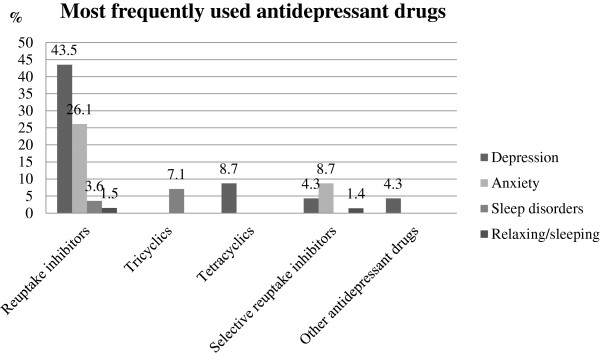
Most frequently used antidepressant drugs to treat the different disorders in this research.

Regarding the type of medicines, these are the most frequently used among drivers interviewed: benzodiazepines to treat anxiety (56.5%), to treat insomnia (35.7%), and depression (26.1%), as tranquilizers (42.6%). The selective serotonin reuptake inhibitors (SSRIs) were used to treat depression (43.5%), anxiety (26.1%), insomnia (3.6%), and as tranquilizers (1.5%) (Figure 4). The table below shows the most frequently used medicines classified according to their specification (Table 5).

**Table 5:** Percentage distribution of medicines used by drivers who were under treatment for different disorders

**Medicines used by drivers who were under treatment for different disorders**	**Percentage**
Drugs to treat depression	
Antidepressant	60.9%
Selective serotonin reuptake inhibitors (SSRI)	(43.5%)
Antidepressant tretracyclics	(8.7%)
Serotonin and noradrenaline reuptake inhibitors	(4.3%)
Other antidepressants	(4.3%)
Anxiolytics	30.4%
Benzodiazepines	(26.1%)
Other anxiolytics	(4.3%)
Hypnotic-sedatives	4.3%
Drugs to treat anxiety	
Anxiolytics	60.9%
Benzodiazepines	(56.5%)
Other anxiolytics	(4.3%)
Antidepressants	34.8%
Selective serotonin reuptake inhibitors (SSRI)	(26.1%)
Serotonin and noradrenaline reuptake inhibitors	(8.7%)
Hypnotic-sedatives	4.3%
Other hypnotic-sedatives	(4.3%)
Drugs to treat insomnia	
Anxiolytics	35.7%
Benzodiazepines	(35.7%)
Hypnotic-sedatives	(35.7%)
Benzodiazepines	(2.9%)
Melatonin receptor agonists	(3.6%)
Imidazopyridines	(5.9%)
Other hypnotic-sedatives	(17.9%)
Antidepressants	10.7%
Selective serotonin reuptake inhibitors (SSRI)	(3.6%)
Tricyclic antidepressants (TCAs)	(7.1%)
Drugs to relax and calm down (tranquilizers)	
Anxiolytics	42.6%
Benzodiazepines	(42.6%)
Hypnotic-sedative	35.7%
Benzodiazepines	(2.9%)
Imidazopyridines	(5.9%)
Other hypnotic-sedatives	(17.6%)
Anti-inflammatory and analgesic drugs	20.6%
Antidepressants	2.9%
Selective serotonin reuptake inhibitors (SSRI)	(1.5%)
Serotonin and noradrenaline reuptake inhibitors	(1.4%)

Distribution of different active principles based on their specific action.

## Discussion

The fact that a high percentage of people (15%) use drugs to treat depression, anxiety, insomnia, and tranquilizers shows that this group of drivers is a risk for road safety since these psychotropic drugs.

These prevalence data are more important if possible, as they are higher than the results of the DRUID project (remember that according to this project, prevalence rate for medicines in Spain was 1.6%. These data would place Spain closest to the group of countries with higher risk as happens in the case of illicit drugs and alcohol.

The distinct methodology used to obtain them is the cause of the differences between the data of the studies (for this section, we use the results of the DRUID project which relate to the prevalence determined by ‘roadside surveys (RSS)’ on drivers and in drivers who have been injured/killed in traffic accidents (hospital studies (HS)).

The fact that 8.3% of those drivers who were not using any drugs to treat these three disorders were occasionally using some type of tranquilizers (7.7% of the total drivers interviewed), may involve a more difficult adaptation (recall that according Berghaus says in a report of the DRUID project, the degree of adaption depends on many factors, especially the dose and the frequency of use).

The fact that more women than men use more drugs to treat depression is also a coinciding data with those determined in the DRUID project on a general level. In the case of the anxiety and tranquilizers in our study, it was not possible to establish a significant relationship between these variables.

It also seems clear that, regarding the age, people between 36–45 use more drugs to treat anxiety and 46–55 use more drugs to depression. Aged 56–65 use drugs to treat insomnia and tranquilizers.

Given the different nature of other studies, it is difficult to make comparisons in the case of age as well as the type of road (roads/cities) and the time of displacement (workday, weekend, holiday, daytime).

Furthermore, other socio-demographic and psycho-social characteristics cannot be contrasted because those have not been contemplated by previous studies.

The fact is that drugs containing benzodiazepine are the most used drugs to treat all the disorders in the study among the drivers interviewed and corresponds with data obtained in the DRUID project and other studies.

However, in our study, the results are also very important on the consumption of different drugs at the same time.

The combined used of drugs for the three conditions studied (depression, anxiety, and insomnia) must also be taken into account since these conditions are usually related to one another thus leading drivers to use medicines for more than one condition (insomnia and anxiety, 29% of the drivers; depression and anxiety, 20%; insomnia and depression, 17%).

## Conclusions

In order to prevent traffic crashes, it is necessary to inform drivers using drugs about the effects they may have on driving and more much control (both the health system and the police).

We propose creating and implementing a wide range of formal intervention strategies. This can be achieved by using general communication campaigns and advertising in order to inform and teach drivers about the influence of several psychotropic drugs on driving.

It is a fact that there are many campaigns on other substances such as alcohol and illicit drugs but much less on psychotropic medications.

The data obtained in this study according to socio-demographic and psychosocial characteristics are very important for the design and dissemination of such campaigns. And it is for both determining the target audience to the communication of certain risk behaviors in Spain.

Standardized warning labels on medicine boxes and package inserts sold in all countries should be implemented as an important countermeasure (have already been implemented in Spain).

Likewise, it is necessary that health professionals (primary health care doctors, health care specialists, pharmacists, psychiatrics, and psychologists) get involved in informing drivers about the side effects (cognitive and psychomotor deterioration) of using psychotropic drugs, as well as the serious consequences of self-medication.

In order to minimize side effects affecting daily activities, it is necessary that health professionals prescribe drugs (specific components, route of administration, avoiding certain associations, and establishing the dose) according to the vital and professional needs of the patient. Some of these demands are related to driving vehicles, specifically when it comes to professional drivers and drivers making daily commutes.

In this sense, the results of this study for Spanish health professionals are very important because socio-demographic and psychosocial characteristics can help identify high risk group. It is also important for police supervision as it can decide better the controls to be made on this behavior both temporally and spatially.

Likewise, it is also necessary to regulate the fact that driving should not be allowed while certain drugs are being used. It would be interesting to authorize doctors to determine, if necessary, those drivers who may be impaired due to the treatment with certain drugs thus lowering road safety.

In this sense, it is very important to establish better communication and collaboration between the health system (hospitals, health centers, etc.) and recognition center conductors (regulated in Spanish Medical and Psychotechnical Exam Model), as the seconds, given the time between recognitions (which only occur in obtaining and renewing the license) cannot detect transient risks such as those that can be derived from the use of these substances.

## Competing interests

The authors declare that they have no competing interests.

## Authors’ contributions

FA elaborated the design of the study with the help of CE; the rest of the authors also contributed. FT and LM were in charge of the data revision. CE also drafted the manuscript. FA performed the statistical analysis. All authors read and approved the final manuscript.

## References

[B1] Ravera S van Rein N de Gier JJ de Jong-van den Berg LT Road traffic accidents and psychotropic medication use in the Netherlands: a case–control study Br J Clin Pharmacol 2011 13 3 505 513 10.1111/j.1365-2125.2011.03994.x 21501214 PMC3175521

[B2] Metzner JL Dentino AN Godard SL Hay DP Hay L Linnoila M Impairment in driving and psychiatric illness J Neuropsychiatry Clin Neurosci 1993 13 2 211 220 8508042 10.1176/jnp.5.2.211

[B3] Gibbons TCN Raffle PAB Mental illness, personality and behaviour disorders Medical Aspects of Fitness to Drive 1976 3 London, England: Medical Commission on Accident Prevention 30 33

[B4] De las Cuevas C Sanz EJ Fitness to drive of psychiatric patients Prim Care Comanion J Clin Psychiatry 2008 13 5 384 390 10.4088/PCC.v10n0506 PMC2629066 19158977

[B5] Harris MC Psychotropic medication and driving Psychiatry Pract 1997 13 5 7

[B6] Odell M Assessing fitness to drive, part 2 Aust Fam Physician 2005 13 6 475 477 15931407

[B7] Montoro L Alonso F Esteban C Toledo F Manual de seguridad vial: El factor humano 2000 Barcelona. Spain: Ariel

[B8] Wang CC Kosinski CJ Schwartzberg JG Shanklin AV Medical conditions and medications that may impair driving Physician’s Guide to Assessing and Counseling Older Drivers 2003 Washington, DC: National Highway Traffic Safety Administration

[B9] Lemoine P Ohayon M Abuse of psychotropic drugs during driving L'Encéphale 1996 13 1 1 6 8681870

[B10] Orriols L Salmi LR Philip P Moore N Delorme B Castot A Lagarde E The impact of medicinal drugs on traffic safety: a systematic review of epidemiological studies Pharmacoepidemiol Drug Saf 2009 13 8 647 658 10.1002/pds.1763 19418468 PMC2780583

[B11] O’Hanlon JF Volkerts ER Hypnotics and actual driving performance Acta Psychiatr Scand 1986 13 332 95 104 10.1111/j.1600-0447.1986.tb08985.x 3554901

[B12] Smiley A Effects of minor tranquilizers and antidepressants on psychomotor performance J Clin Psychiatry 1987 13 22 28 2891686

[B13] Robbe H Marijuana’s impairing effects on driving are moderate when taken alone but severe when combined with alcohol Hum Psychopharm Clin 1998 13 2 70 78

[B14] Kelly E Darke S Ross J A review of drug use and driving: epidemiology, impairment, risk factors and risk perceptions Drug Alcohol Rev 2004 13 319 344 10.1080/09595230412331289482 15370012

[B15] Engeland A Skurtveit S Morland J Risk of road traffic accidents associated with the prescription of drugs: a registry-based cohort study Ann Epidemiol 2007 13 8 597 602 10.1016/j.annepidem.2007.03.009 17574863

[B16] Movig KL Mathijssen MP Nagel PH van Egmond T de Gier JJ Leufkens HG Egberts AC Psychoactive substance use and the risk of motor vehicle accidents Accid Anal Prev 2004 13 4 631 636 10.1016/S0001-4575(03)00084-8 15094417

[B17] Verster JC Mets MA Psychoactive medication and traffic safety Int J Environ Res Public Health 2009 13 3 1041 1054 10.3390/ijerph6031041 19440432 PMC2672393

[B18] Smink BE Egberts AC Lusthof KJ Uges DR de Gier JJ The relationship between benzodiazepine use and traffic accidents: a systematic literature review CNS Drugs 2010 13 8 639 653 10.2165/11533170-000000000-00000 20658797

[B19] Schulze H Schumacher M Urmeew R Auerbach K DRUID Final Report: Work Performed, Main Results and Recommendationshttp://www.druid-project.eu

[B20] Hargutt V Krüger H-P Knoche A Driving Under the Influence of Alcohol, Illicit Drugs and Medicines. Risk Estimations from Different Methodological Approaches DRUID deliverable 1.3.1 2011 http://www.druid-project.eu

[B21] Berghaus G Sticht G Grellner W Lenz D Naumann T Wiesenmüller S Meta-Analysis of Empirical Studies Concerning the Effects of Medicines and Illegal Drugs Including Pharmacokinetics on Safe Driving 2010 EU Project DRUID, WP 1, Deliverable 1.1.2b

[B22] Gee GC Takeuchi DT Traffic stress, vehicular burden and wellbeing: a multilevel analysis Soc Sci Med 2004 13 405 414 10.1016/j.socscimed.2003.10.027 15110429

[B23] Alonso F Esteban C Calatayud C Alamar B Egido A Salud Vial. Teoría y prácticas de los trastornos físicos y psíquicos en la conducción. Cuadernos de Reflexión Attitudes 2008 Valencia, Spain: Intras

[B24] Alonso F Sanmartín J Esteban C Calatayud C Alamar B López E Salud Vial. Diagnóstico de los conductores españoles. Cuadernos de Reflexión Attitudes 2008 Valencia, Spain: Intras

[B25] Alonso F Esteban C Calatayud C Alamar B Fernández C Medina JE Conclusiones de las 7as jornadas de reflexión Attitudes: Salud vial ¿el conductor a terapia? Cuadernos de Reflexión Attitudes 2008 Valencia, Spain: Intras

[B26] Sánchez F Actitudes frente al riesgo vial Intervención Psicosocial 2008 13 1 45 59

